# Using machine learning to predict depression among middle-aged and elderly population in China and conducting empirical analysis

**DOI:** 10.1371/journal.pone.0319232

**Published:** 2025-03-18

**Authors:** Zhe Wang, Ni Jia

**Affiliations:** 1 Department of Public Health, Shaanxi University of Chinese Medicine, Xianyang, Shaanxi, China; 2 First Clinical Medical College, Shaanxi University of Chinese Medicine, Xianyang, Shaanxi, China; University of Roehampton - Digby Stuart College, UNITED KINGDOM OF GREAT BRITAIN AND NORTHERN IRELAND

## Abstract

**Objective:**

To develop a predictive model for evaluating depression among middle-aged and elderly individuals in China.

**Methods:**

Participants aged ≥ 45 from the 2020 China Health and Retirement Survey (CHARLS) cross-sectional study were enrolled. Depressive mood was defined as a score of 10 or higher on the CESD-10 scale, which has a maximum score of 30. A predictive model was developed using five selected machine learning algorithms. The model was trained and validated on the 2020 database cohort and externally validated through a questionnaire survey of middle-aged and elderly individuals in Shaanxi Province, China, following the same criteria. SHapley Additive Interpretation (SHAP) was employed to assess the importance of predictive factors.

**Results:**

The stacked ensemble model demonstrated an AUC of 0.8021 in the test set of the training cohort for predicting depressive symptoms; the corresponding AUC in the external validation cohort was 0.7448, outperforming all base models.

**Conclusion:**

The stacked ensemble approach serves as an effective tool for identifying depression in a large population of middle-aged and elderly individuals in China. For depression prediction, factors such as life satisfaction, self-reported health, pain, sleep duration, and cognitive function are identified as highly significant predictive factors.

## 1 Background introduction

Depression is a prevalent mental health issue among the middle-aged and elderly population, associated with a myriad of adverse health outcomes, including impaired sleep quality [1],cognitive decline [2],and even an elevated risk of all-cause mortality [3]. The Center for Epidemiological Studies Depression Scale (CES-D), a self-rating scale, is currently the most widely utilized tool for measuring and evaluating depression in large-scale epidemiological studies [4]. The CES-D assesses the frequency of various emotional experiences, such as feelings of depression, happiness, and loneliness, as reported by respondents over the past week, based on personal emotional changes. As the population ages, the burden of depression on the middle-aged and elderly is also escalating [5].In the context of rapid population aging, early identification and risk assessment of depressive emotions are imperative.

Many studies have delved into the specific risk factors that influence depression, including cardiovascular disease [6],Insomnia disorder [7], urbanization [8],and chronic diseases [9]. Some research has employed more traditional regression models in conjunction with multidimensional domain variables [10,11]. However, the underlying principles of traditional logistic regression methods are relatively straightforward, and the statistical procedures can be somewhat simplistic. These methods often rely on the assumption that there is a linear relationship between independent and dependent variables. In contrast, contemporary machine learning techniques are more sophisticated and capable of handling high-dimensional data, thereby aiding in the discovery of potential causal patterns. In recent years, the application of machine learning across various disciplines has seen rapid growth, particularly in the field of disease prediction [12–14].

While previous studies have explored the application of machine learning algorithms for depression prediction among specific populations such as elderly individuals with cognitive impairments [15] or epilepsy patients [16], there remains a significant gap in research focusing on the general population. Our study addresses this gap by developing a machine learning-based depression prediction model that is tailored to middle-aged and elderly individuals without specific health conditions, thereby providing a more generalized and widely applicable tool for depression prediction. Furthermore, the lack of consensus on the most effective algorithm or model configuration is alleviated in our research through a comparative analysis of five selected machine learning algorithms. We have identified a stacked ensemble model as the most predictive, offering a novel approach to depression prediction that outperforms individual algorithms. This is particularly significant given the paucity of integrated machine learning models developed specifically for depression. Lastly, our study is among the few to conduct field testing of a machine learning model trained on big data for depression prediction. By externally validating our model through a questionnaire survey in Shaanxi Province, China, we provide empirical evidence of the model’s effectiveness in a real-world setting, contributing to the field by demonstrating the practical application and generalizability of our approach.

Thus, the aim of this study is to leverage data from the China Health and Retirement Longitudinal Study (CHARLS) [17]to develop appropriate machine learning models that can identify individuals at risk of developing depression in the future. We plan to validate these models within the province. Our study aims to contribute to a more nuanced understanding of depression prediction and to provide a robust, empirically validated model that could be applied in both clinical and public health settings. We believe that our approach not only advances the field but also offers a valuable resource for future research endeavors. CHARLS is a nationally representative longitudinal study focusing on adults aged 45 and older from 28 provinces across China, providing a robust dataset for our analysis.

## 2 methods

### 2.1 Research sample

This research utilized data from the 2020 China Health and Retirement Longitudinal Study (CHARLS). The survey aims to gather comprehensive information and research data on middle-aged and elderly individuals aged 45 and over, as well as their families in China, and is frequently employed in the field of aging population research within the country. The CHARLS questionnaire is designed with reference to the layout principles of internationally renowned surveys, ensuring a rich and diverse content that encompasses representative and thorough data on the middle-aged and elderly population and their families in China. CHARLS officially initiated data collection in 2011, with a biennial or triennial tracking interval. To date, five national tracking surveys have been completed in 2011, 2013, 2015, 2018, and 2020, with corresponding follow-up data being released. The survey now covers 28 provinces across China, including hundreds of village and county-level units, with a cumulative sample size of 12,400 households and 190,000 individuals. CHARLS is esteemed for its wide coverage, well-designed questionnaire structure, and relatively high response rate and data quality, which are on par with global standards in related field surveys. Its follow-up data hold significant research value and are highly favored and widely applied by scholars both domestically and internationally. The CHARLS study has received ethical approval from the Biomedical Ethics Review Committee of Peking University. All participants provided written informed consent, ensuring the integrity and ethical standards of the research.

Furthermore, this study complemented the national dataset with random surveys conducted in Shaanxi Province, China from December 2022 to February 2024, employing methods such as questionnaire administration and interviews to collect over 1,000 data points relevant to the research. All participants provided written informed consent, ensuring the integrity and ethical standards of the research. This study was approved by the Ethics Committee of the Affiliated Hospital of Shaanxi University of Chinese Medicine, and it was deemed to meet the requirements for ethical exemption, thus waiving the need for ethical review.

**Fig 1:**
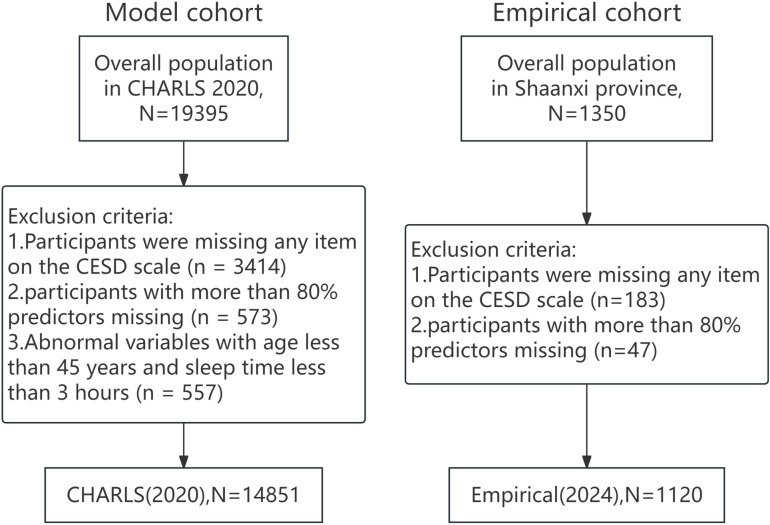
Subject selection flowchart.

The data exclusion process for this study is illustrated in Fig 1. We selected the 2020 dataset (n = 19,395) from the database to serve as the training cohort. The exclusion criteria for the model cohort were as follows: (1) Any participant with missing items in the CESD scale (n = 3,414); (2) Rows with less than 10% missing values for a given variable (n = 573); (3) Abnormal cases with age below 45 years and sleep duration less than 3 hours (n = 557). Consequently, a total of 14,851 individuals were available for model construction and validation.

For the empirical cohort, the exclusion criteria included: (1) Participants missing any items in the CESD scale (n = 183); (2) Participants with more than 80% missing predictor variables (n = 47). As a result, 1,120 individuals were eligible for the empirical testing of the model.

### 2.2 Data collection

Drawing on existing literature and subjective insights, we identified 30 secondary indicators across six domains that potentially influence depression in the middle-aged and elderly population. These indicators include: (1) sociodemographic characteristics: gender, age, marital status, urban-rural residence, and educational level; (2) subjective self-assessment factors: self-rated satisfaction with child relationships and self-rated life satisfaction; (3) individual factors: disability, medication or treatment status, physical pain, daily sleep duration, social activities in the past month, current or past smoking, current or past alcohol consumption, and difficulties with daily activities; (4) economic factors: presence of a salary, family business ownership, and monthly consumption; (5) chronic illnesses: hypertension, diabetes, cancer, lung disease, heart disease, stroke, mental health issues, arthritis, dyslipidemia, liver disease, kidney disease, digestive system disorders, asthma, and Alzheimer’s disease; (6) social security factors: medical insurance status and pension insurance status. After careful consideration, 24 predictive factors were retained for analysis to address the research questions.

### 2.3 Result determination

In the CHARLS study, we utilized the CES-D scale, developed by Radloff in 1977 [18],and adapted the simplified CESD-10 scale used by subsequent scholars to assess depression status based on self-reported data. The CESD-10 scale consists of 10 items: “Worry,” “Difficulty Concentrating,” “Low Mood,” “Effort to Do Things,” “Hope for the Future,” “Fear,” “Insomnia,” “Happiness,” “Loneliness,” and “Unable to Carry On.” Within CHARLS, the CESD-10 measures the frequency of these feelings experienced by respondents over the past week. Scoring is based on the frequency of each feeling: less than one day is termed “none or almost none,” scoring 0 points; 1-2 days is “rare,” scoring 1 point; 3-4 days is “frequent,” scoring 2 points; and 5-7 days is “almost all,” scoring 3 points. Two of these questions pertain to positive emotions and thus require reverse scoring (e.g., 3 points for less than one day, and so on). The total score from all 10 questions is summed, resulting in a range of 0-30 points. A total score of 10 or higher is indicative of depressive mood, while a lower score suggests the absence of depressive mood. Moreover, the higher the total score, the more severe depression experienced by the interviewee.

### 2.4 Statistical analysis

**Fig 2:**
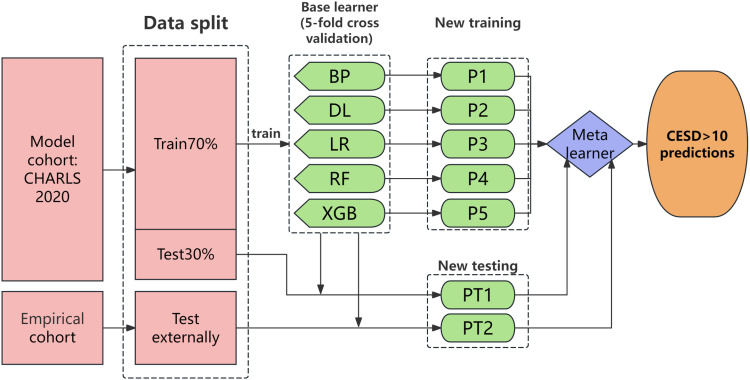
Architecture of model construction. BP, BP neural network; DL, deep learning; LR, logistic regression; RF, random forest; XGB, XGBoost.

In the preprocessing of baseline covariates, continuous variables were normalized using the min-max scaling method, where values were scaled to a range between 0 and 1. Categorical variables were processed through one-hot encoding. Missing values in categorical variables were imputed using the median imputation method, while missing values in numerical variables were estimated using the random forest technique [19]. In the preprocessing of baseline covariates, median imputation was selected for categorical variables to preserve the distributional characteristics without the assumption of a normal distribution and to mitigate the influence of potential outliers. For numerical variables, the random forest imputation technique was employed due to its capability to capture complex, non-linear relationships within the dataset, thereby providing more accurate estimations of missing values compared to traditional imputation methods. These choices were made to ensure the robustness of our analysis and the reliability of our results, given the specific characteristics of our dataset and the objectives of our study. The dataset of 14,851 entries was divided into training and testing sets at a ratio of 7:3, with the detailed modeling process depicted in Fig 2.

**Fig 3:**
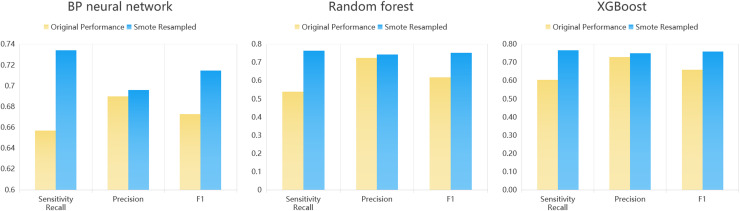
Comparative analysis of evaluation metrics for three models before and after SMOTE resampling.

Within this dataset, 5,099 participants (34.33%) exhibited depressive symptoms. To address potential data imbalance, the Synthetic Minority Oversampling Technique (SMOTE) [20]was applied to the training set to oversample the dataset in a targeted manner. We identified the minority class (depressed individuals) and determined the number of synthetic samples needed to balance the dataset. Using the K-nearest neighbors algorithm, we generated synthetic samples by interpolating between existing minority class instances. These results, presented in Fig 3 and Table 1, highlight SMOTE’s effectiveness in improving model performance metrics, particularly sensitivity and F1 score. Overall, the SMOTE technique enhanced the performance of all models, particularly in terms of sensitivity and F1 score. These results underscore the importance of addressing class imbalance in improving the predictive accuracy of machine learning models for depression detection.

**Table 1:** Performance Evaluation of Three Models Before and After SMOTE Resampling.

Model		Sensitivity Recall	Precision	F1
BP neural network	Original Performance	0.6568	0.6897	0.6728
	Smote Resampled	0.7342	0.6959	0.7145
Random forest	Original Performance	0.5398	0.7262	0.6193
	Smote Resampled	0.7649	0.7428	0.7537
XGBoost	Original Performance	0.6032	0.7296	0.6604
	Smote Resampled	0.7679	0.7503	0.7590

In the feature selection process, the Akaike Information Criterion (AIC) was utilized as a criterion for model selection. AIC is a measure based on the likelihood function that is used to estimate the relative amount of information lost by a given model. It balances the trade-off between the goodness of fit of the model and the complexity of the model, penalizing models with too many parameters. By employing AIC, we aimed to identify the optimal combination of model features that would best predict the outcome while avoiding overfitting. The stepwise regression algorithm, incorporating AIC, was used to iteratively add or remove predictors, thereby effectively screening features and mitigating the impact of multicollinearity issues [21]. The final variable combination was determined to consist of 24 features, with the specific variables and their correlation levels detailed in Table 2.

**Table 2:** The specific variables and their correlation levels.

Variable	df	AIC	Estimate	P
Stroke	1	26430	-0.03916	0.124
Heart disease	1	26430	-0.02627	0.094
Have Wage	1	26429	0.01602	0.058
businesses	1	26429	0.02011	0.057
Drink	1	26428	0.00920	<0.05
Consumption	1	26423	-0.00003	<0.01
Mental	1	26423	-0.11071	<0.01
Frequency_see	1	26422	0.00478	<0.01
ADL	1	26422	0.01077	<0.001
Use Internet	1	26421	0.02864	<0.001
Alzheimer	1	26418	-0.07727	<0.001
Education	1	26416	-0.00937	<0.001
Age	1	26414	-0.00206	<0.001
Marriage	1	26405	0.01430	<0.001
Place	1	26391	-0.03511	<0.001
Cognitive	1	26388	-0.02024	<0.001
disability	1	26384	-0.04564	<0.001
difficult	1	26383	0.03909	<0.001
Chidren Satisfication	1	26377	0.03802	<0.001
Gender	1	26368	0.06614	<0.001
self reported health	1	26311	0.04396	<0.001
Sleeptime	1	26301	-0.02116	<0.001
Pain	1	26223	0.04611	<0.001
Life satisfication	1	26099	0.09023	<0.001

The training cohort was optimized using five-fold cross-validation to select the best parameters, and the performance of the model debugging was assessed by the area under the curve (AUC) in the training set. Detailed hyper-parameters of each model were listed in supplementary material S1 Table. A larger AUC indicates superior model performance. Additionally, we employed other metrics to evaluate the model’s performance, such as accuracy, sensitivity, specificity, and F1 score. The dataset was subjected to nine machine learning algorithms, including support vector machines, BP neural networks, deep learning, logistic regression, random forests, XGBoost, KNN, gradient boosting machines, and AdaBoost. Based on the average performance of each algorithm on the training set, we excluded those models with AUC values below 0.7.Ultimately, our chosen machine learning model includes: BP neural network, Deep Learning, Logistic

Regression, Random Forest and XGBoost. The specific performance of the nine models is provided in the Supplementary Material S2 Table.

Following the feature selection process, the surviving models were integrated using a stacked ensemble approach. This method, also known as stacking, is a machine learning technique that combines multiple base classifiers to enhance predictive accuracy. The stacked ensemble model comprises two layers of estimation algorithms. The first layer, known as the base layer, consists of individual classifiers trained on the training data using a variety of traditional machine learning algorithms. These classifiers may include, but are not limited to, logistic regression, decision trees, and support vector machines. The second layer, referred to as the meta layer, consists of one or more meta classifiers that take the predictions from the base classifiers as input features and make the final prediction. This hierarchical structure allows the stacked ensemble model to leverage the strengths of multiple classifiers and to correct for their individual weaknesses, thereby improving the overall performance of the model. In the present study (refer to Fig 2), we developed a BP neural network that incorporates five classifiers: Deep Learning (DL), Logistic Regression (LR), Random Forest (RF), and XGBoost. We conducted five-fold cross-validation to create the training set for the meta-level model. At the meta level, an LR classifier was utilized.

Subsequently, we employed the SHapley Additive Interpretation (SHAP) method to assess the importance of various predictive factors [22].SHAP is grounded in cooperative game theory and provides a means to quantify the impact of each feature on the model’s predictions. It does so by computing the Shapley value for each feature, which represents the average marginal contribution of that feature to the model’s output across all possible coalitions of features. In essence, SHAP evaluates the importance of a specific variable by comparing the model’s predictions with and without the variable in question, thus providing a measure of the variable’s influence on the prediction. The SHAP method not only provides a qualitative assessment of feature importance but also allows for a visual representation that aids in understanding the complex interactions within the model. Additionally, we directly evaluated the importance of the top 15 variables in each machine learning model. All statistical analyses were performed using R software (4.4.1).

Finally, the five well-trained base models and the ensemble training model will be subjected to empirical analysis on 1,120 real-world data points collected within Shaanxi Province. The empirical data were meticulously gathered according to the variables necessitated by the training set, ensuring consistency in dataset processing and standardization the training set. This approach allowed us to obtain empirical validation curves for each model, which were then compared to assess their performance in a real-world context and to address the research questions at hand.

## 3 Results

### 3.1 Baseline characteristics

The baseline characteristics of the dataset used to train the model are presented in Table 3. Within the training cohort, there were a total of 14,851 middle-aged and older participants. According to the statistical data from 2020, the average age of the depressive population was younger, and there was a higher prevalence of depression among women compared to men. The characteristics of the depressive population often included lower levels of education, a higher likelihood of a history of stroke, a predominantly agricultural household registration status, limited internet literacy, poor cognitive function, and an extremely low self-rated satisfaction with life.

**Table 3:** Selected baseline characteristics of participants.

Variable		Depression (5099)	Non depressed (9752)	P
		CESD ≥ 10	CESD < 10	
Age (year)		60.24	61.22	<0.001
Gender	male	1890	5376	<0.001
	female	3209	4376	
Education	Not receiving education	1202	1484	<0.001
	Elementary school and below	2473	3991	
	junior high school	1004	2629	
	high school	306	1058	
	undergraduate	99	485	
	master	13	96	
	doctor	2	9	
marital status	married	4286	8638	<0.001
	separation	25	38	
	divorce	68	134	
	Widowed	714	934	
	unmarried	6	8	
drink wine	Drink frequently	1063	3131	<0.001
	Occasionally drink	471	1018	
	Not drinking	3565	5603	
Stroke	yes	158	115	<0.001
	no	4941	9637	
Place	Agriculture	4137	6843	<0.001
	Non agricultural	561	1747	
	other	401	1162	
Use internet	yes	1862	4770	<0.001
	no	3237	4982	
cognition	difference	499	552	<0.001
	secondary	1837	2405	
	good	2763	6795	
Life satisfaction	Extremely satisfied	153	554	<0.001
	Very satisfied	1081	3512	
	Quite satisfied	2774	5287	
	Not very satisfied	838	313	
	dissatisfied	253	86	

### 3.2 Model performance

**Fig 4:**
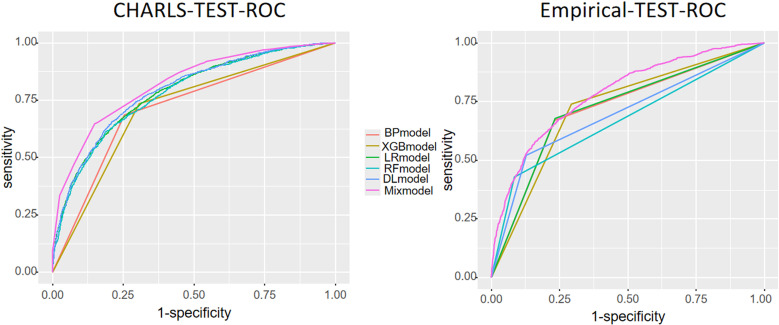
Performance curves of various models.

The performance metrics of each model are detailed in Table 4 and visually represented in Fig 4. The stacked ensemble model exhibited the superior discriminative power in predicting depression status on both the CHARLS test dataset and the empirical dataset, with Area Under the Receiver Operating Characteristic Curve (AUC) values of 0.8021 and 0.7448, respectively. In terms of F1 score, the stacked ensemble model was slightly outperformed by the deep learning model on the CHARLS test dataset (0.7872), but it demonstrated the highest F1 score among all models on the empirical test dataset, reaching 0.7787 and 0.7625, respectively. Furthermore, the stacked ensemble model also showed the best accuracy performance on both datasets, with accuracy rates of 0.7872 and 0.7660.

**Table 4:** Performance of each model.

Model		Accuracy	Sensitivity	specificity	AUC	Precision	F1
BP neural network	CHARLS data	0.7232	0.7342	0.6792	0.7442	0.6959	0.6825
	Empirical data	0.6821	0.6891	0.6421	0.6696	0.6582	0.6733
Deep learning	CHARLS data	0.7764	0.8035	0.7621	0.7932	0.7716	0.7872
	Empirical data	0.7284	0.7356	0.7025	0.7128	0.7120	0.7236
Logistic regression	CHARLS data	0.7570	0.7764	0.7072	0.7860	0.7262	0.7504
	Empirical data	0.7331	0.7343	0.7159	0.7023	0.7210	0.7276
Random forest	CHARLS data	0.7568	0.7649	0.7352	0.7761	0.7428	0.7537
	Empirical data	0.7146	0.7139	0.7201	0.6866	0.7184	0.7161
XGBoost	CHARLS data	0.7498	0.7679	0.7445	0.7546	0.7503	0.7590
	Empirical data	0.7284	0.7334	0.6847	0.6932	0.6993	0.7160
Mix model	CHARLS data	0.7872	0.7923	0.7575	0.8021	0.7657	0.7787
	Empirical data	0.7660	0.7859	0.7245	0.7448	0.7404	0.7625

When comparing individual models, the logistic regression and random forest models performed better on the CHARLS dataset test set, achieving AUC values of 0.7860 and 0.7761, respectively. However, their performance was suboptimal on the empirical test set, with AUC values of only 0.7023 and 0.6866, indicating poor generalization ability. The deep learning model achieved the highest performance on the CHARLS test set with an AUC of 0.7932 and also performed the best on the empirical test set with an AUC of 0.7281, suggesting strong stability and adaptability of the deep learning model. Additionally, the deep learning model boasted high F1 scores, reaching 0.7872 and 0.7236, respectively, on the two datasets.

### 3.3 Variable importance analysis

Upon training various models, the importance of variables within the four fundamental models, excluding deep learning models, was quantified using SHAP values. This information is depicted through bar charts and honeycomb plots for visual comparison. The machine learning model leveraged the ‘varimp’ function provided by the H2O platform to present its variable importance bar chart, thereby mitigating potential calculation biases.

In the context of this research, self-assessment of life satisfaction emerged as the most influential variable affecting depression status, occupying the top rank across all models. However, the ranking of the remaining variables differed among the various models. Variables that consistently ranked highly included self-rated health status, physical pain, difficulty in performing tasks, total sleep time, and issues related to disability.

**Fig 5:**
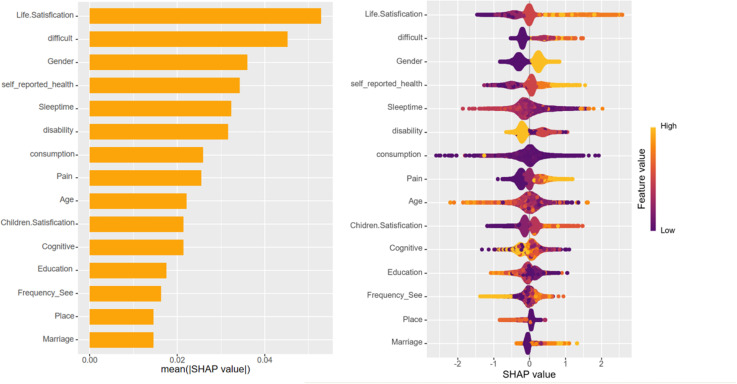
Bar chart and honeycomb plot of the top 15 variable features in BP neural network model.

**Fig 6:**
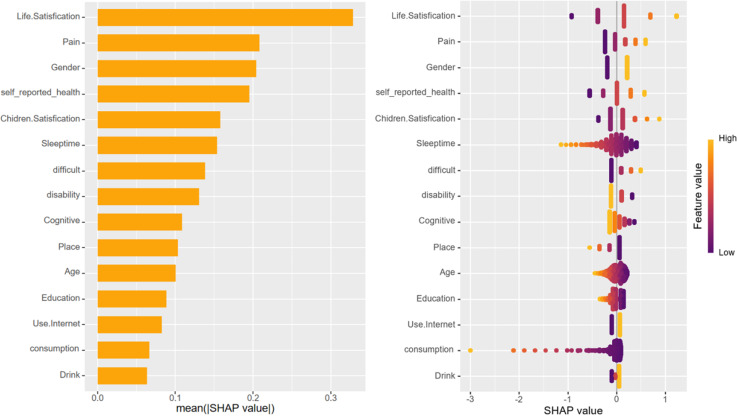
Bar chart and honeycomb plot of the top 15 variables in Logistic Regression model.

**Fig 7:**
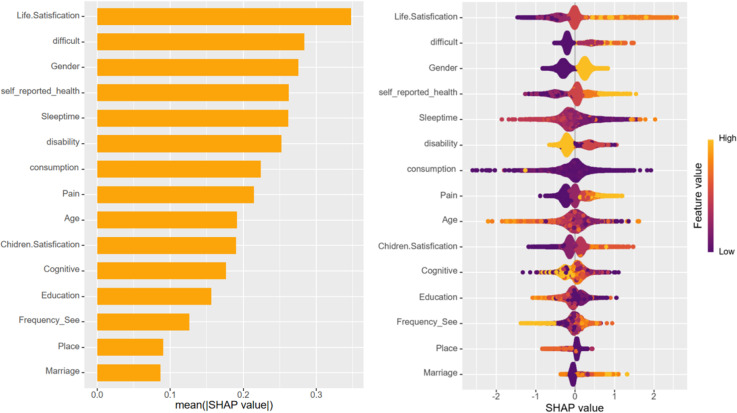
Bar chart and honeycomb plot of the top 15 variables in the XGBoost model.

**Fig 8:**
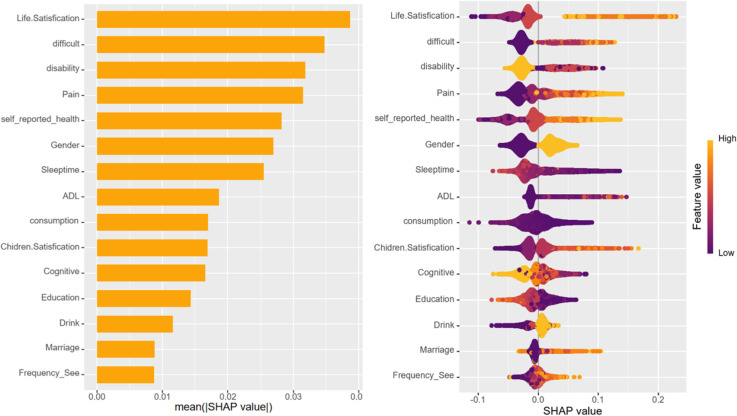
Bar chart and honeycomb plot of the top 15 variables in the Random Forest model.

**Fig 9:**
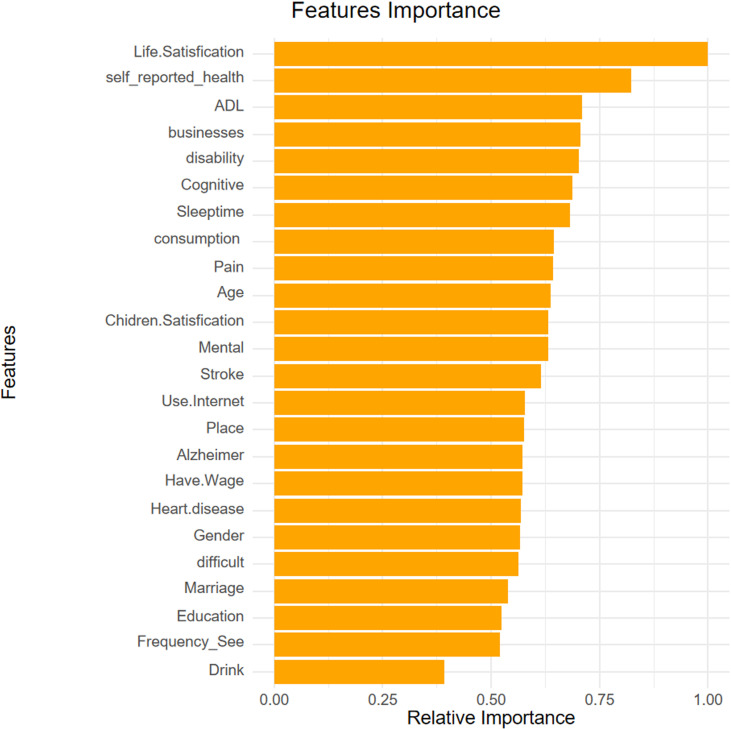
Explanation of variables in varimp function of Deep Learning.

In the case of deep learning models, variables such as dependency issues and whether a family owns a business were found to contribute more significantly to cognitive scenarios, setting them apart from the other models in terms of their impact on depression status (Figs 5–9).

## 4 Discussion

In the present study, we constructed a machine learning prediction model designed to identify adults at elevated risk of functional dependence using advanced machine learning techniques. Moreover, our findings indicate that individuals who report low life satisfaction, poor self-perceived health, severe physical pain, or difficulties in performing various activities are at a heightened risk of developing depressive symptoms in the near future.

Prior research has frequently relied on regression analysis and basic machine learning algorithms for the prediction of depressive symptoms. For instance, one study [23]collected data from 15,661 participants aged 50 and over from the U.S. Health and Retirement Study. Major depression was assessed using Composite International Diagnostic Interview Short Form (CIDI-SF). The study employed a population-based trajectory model to delineate depression trajectories and to elucidate the concept of non-random attrition. Predictors of depression trajectories were investigated using multiple logistic regression analysis. The findings revealed that individuals aged 50 and above, males, and non-Hispanic African Americans had a lower risk for trajectories with low to high levels of depression burden, while the count of chronic diseases was associated with an increased risk of such trajectories. Additionally, the risk for trajectories characterized by a constant increase in depression symptoms rose in conjunction with the difficulty of action. The presence of difficulties in family activities signals a persistent increase in the number of individuals within the middle-to-old age bracket. Shao et al. [24]conducted a screening of 16,512 participants identified as meeting the inclusion criteria from the China Health and Retirement Longitudinal Study (CHARLS) Database. Utilizing a variety of models based on potential predictive factors, they employed stepwise regression (logistic regression) and random forest models to explore the influence and weights of candidate factors on depression risk. Their findings highlighted gender, place of residence, changes in health status since the last interview, physical disability, chronic pain, child health status, activities of daily living, and social activities as independent risk factors for depression. Among these factors, chronic pain exerted a particularly significant impact on the likelihood of depression. Similarly, Rong et al. [25]leveraged the CHARLS dataset to predict the depression status of the elderly population, employing both traditional regression methods and random forest algorithms to achieve relatively precise models, with AUC values ranging from 0.769 to 0.795. The key determinants identified were life satisfaction, self-reported memory, cognitive ability, and disorders in activities of daily living. Life satisfaction was found to increase the risk of future depression by 128.6%, 13.8%, and 13.2% respectively, whereas cognitive ability emerged as the most crucial predictor within the random forest framework. In recent years, the application of machine learning techniques for predictive analytics has gained substantial traction and is increasingly acknowledged as a potent statistical tool [26]. Our study’s machine learning algorithm surpasses traditional regression models by identifying intricate relationships among various variables. Consequently, this model possesses, in theory, a higher discriminative power compared to its traditional counterparts.

For instance, a seminal study utilized outbreak data from 43 diseases across 206 countries to construct a universal risk prediction system, which can be applied to diverse nations and diseases [27]. This system integrates five machine learning models—including the neural network-based XGBoost model, logistic boosting model, random forest model, and kernel support vector machine model—for predictive analytics and employs a voting mechanism for overall prediction. It is capable of generating forecasts from a multifaceted perspective that encompasses economic, cultural, social, and epidemiological factors, achieving an accuracy rate of approximately 80% to 90%. To assess the performance of machine learning models in various practical scenarios, three distinct datasets were designed. The prediction system demonstrated robust predictive power, adaptability, and versatility, marking it as a promising approach for future applications in health and beyond. The proposed model is capable of conducting comprehensive epidemic risk assessments that transcend geographical borders and disease specificity, thereby facilitating swift responses to pandemics, informing governmental decision-making processes, and enhancing international collaborative efforts. In alignment with this objective, our study aims to perform empirical validation across various provinces within China. The model has exhibited a commendable predictive recognition capability in both the training and validation datasets, thereby providing substantial evidence of its stability and reliability within the context of this investigation.

Certainly, to further ascertain the efficacy of the model’s application, an expansion of the sample size is essential. While the current research is grounded in surveys conducted within China, the methodologies employed for model development are adaptable and can be extended to diverse populations, including those of different racial backgrounds or age groups. This flexibility suggests that the model’s utility is not limited to the specific context of China and holds the potential for broader application in addressing global health challenges. By doing so, we can contribute to a more nuanced understanding of epidemic dynamics and improve preparedness and response strategies on a worldwide scale.

In this investigation, we developed a predictive model for depression status based on the CES-D scale. Through Akaike Information Criterion (AIC) feature selection, we identified 25 pertinent variables and constructed a stacked ensemble model, which exhibited robust predictive performance in the CHARLS test cohort. Subsequently, we collected and processed real-world data from middle-aged and elderly individuals in Shaanxi, China, using the same methodology, and applied these data to our model for predictive analysis. Despite a decrease in AUC, the model retained impressive predictive capabilities, and the number of variables involved remains manageable for clinical surveys.

To elucidate the contribution of the top 15 variables in certain foundational models, we utilized SHAP (SHapley Additive Explanations) bar charts and honeycomb plots. These visualizations employ color coding to represent variable value levels, with orange indicating higher values and purple denoting lower values. The thickness of lines composed of individual points corresponds to the sample size at each value, providing a quantitative measure of the contribution of each variable to the model’s predictions. The x-axis charts the influence of variables on the outcomes, where a positive SHAP value signifies an elevated risk, whereas a negative SHAP value denotes a reduced risk. For instance, in the context of predicting life satisfaction, a higher value of life satisfaction corresponds to greater dissatisfaction with life and, accordingly, an increased risk of developing depression.

While our predictive model holds significant promise for forecasting depression and dependence in the foreseeable future, it is imperative to refine its application across various scenarios and to tailor its use in clinical settings and institutional frameworks within the diverse landscape of healthcare. Clinicians and professionals in related fields must also receive adequate training to interpret the model’s results accurately and appropriately.

The generalizability of our predictive model to other regions or countries is an important consideration. While our external validation was conducted within Shaanxi Province, China, we acknowledge that the model’s performance may be influenced by regional differences in socio-economic status, healthcare access, and cultural factors. To address this, we have conducted a thorough analysis of the model’s robustness by examining how the identified predictive factors correlate with depression across diverse settings. Our findings indicate that the key predictive factors—life satisfaction, self-reported health, pain, sleep duration, and cognitive function—are well-documented risk factors for depression that are likely to be relevant in other middle-aged and elderly populations, both within China and internationally. Furthermore, we have explored the model’s sensitivity to variations in these factors and have identified potential adjustment strategies that could enhance its applicability to different socio-economic and healthcare contexts. These strategies include incorporating region-specific variables and adapting the model to account for cultural differences in the expression of depressive symptoms. Future research should focus on validating the model in additional regions and countries to further assess its generalizability and to refine it for broader use. In the present investigation, we identified life satisfaction as a critical predictor of depression risk, a finding that aligns with the literature [28,29]. Specifically, a higher level of life satisfaction is associated with a reduced likelihood of developing depression. Our findings are in concordance with prior research indicating a distinct link between sleep duration and the incidence of depression [30]. Furthermore, self-reported health status has been validated as a significant predictor of depressive symptoms, corroborating the results observed in our study [31]. In synergy with earlier studies, we also found that persistent pain serves as a pivotal predictor in the context of depression, a finding that is supported by Mendelian randomization studies [32]. A positive outlook on life, adequate sleep duration, and healthy physical condition emerged as key predictive factors for the occurrence of depressive symptoms. Our research further validates that the extent of disability is an influential determinant of depression risk [33]. Within the framework of our basic model, cognitive ability plays a substantial role in predictive accuracy. Consequently, it is imperative to closely monitor the mental health of middle-aged and elderly individuals who exhibit compromised cognitive function [34].

The current study presents several notable limitations that warrant careful consideration. Firstly, the machine learning model we have developed is specifically tailored to the middle-aged and elderly population, which inherently restricts the generalizability of our findings to other age groups. This limitation underscores the need for future research to validate and adapt the model for broader demographic application. Secondly, the empirical analysis conducted on a sample of middle-aged and elderly individuals in Shaanxi province is susceptible to various statistical biases, including those related to sample size, data preprocessing, and feature selection. These factors can significantly influence the model’s performance and its practical applicability. It is essential to acknowledge that the results may be influenced by these methodological choices and should be interpreted with caution. Thirdly, the dataset utilized in our study, which is based on the 2020 research survey, lacks additional physiological and biochemical indicators that could potentially serve as important predictive factors for depression. The absence of such data limits our ability to explore a comprehensive range of variables that may contribute to the risk of depression. Furthermore, it is important to note that depressive states can be dynamic and subject to change time. The cross-sectional nature of our predictive model does not capture the temporal evolution of depression.

Despite the identified limitations, our study possesses several significant strengths. Firstly, it is grounded in a nationally representative cohort characterized by a substantial sample size and a comprehensive array of predictive factors. This robust foundation enhances the reliability and relevance of our findings. Secondly, we have crafted a precise and user-friendly tool for forecasting the risk of functional dependence among middle-aged and elderly individuals in China. This innovative tool has the potential to enhance clinical and health management practices by enabling the identification of individuals at high risk, thereby facilitating timely interventions and improving patient outcomes. Lastly, our research leveraged a model that has been refined through big data analytics for practical validation and analysis. The model has demonstrated impressive adaptability, suggesting that it can be effectively applied in diverse clinical settings. This adaptability, along with the insights gained from our analysis, holds the promise of contributing valuable knowledge to future clinical assessments and health-related decision-making processes.

In conclusion, while acknowledging the limitations of our study, we believe that the advantages outlined above position our research to make a meaningful impact on the understanding and management of depression in the middle-aged and elderly population. The findings from this study can serve as a foundation for further investigation and refinement of the model, potentially leading to more sophisticated tools that can be integrated into routine clinical practice.

## 5 Conclusion

In this study, we have successfully developed a machine learning model tailored for predicting depression symptom among a substantial cohort of middle-aged and elderly adults in China. The utilization of stacked ensemble models has proven to be an efficacious approach for not only Anticipating the trajectory of depressive symptoms but also for evaluating the risk of depression onset. Such models hold the potential to serve as a valuable resource for communities, hospitals, and a broad spectrum of institutions and populations in the provision of fundamental mental health care services.

## Supporting information

S1 Table Hyperparameter settings of each models.
(XLSX)

S2 Table Performance of nine models.
(XLSX)

S3 Table Empirical data.
(XLSX)
